# Association between systemic immune inflammation Index and all-cause mortality in incident peritoneal dialysis-treated CKD patients: a multi-center retrospective cohort study

**DOI:** 10.1186/s12882-023-03451-4

**Published:** 2024-01-03

**Authors:** Ruiying Tang, Jiexin Chen, Qian Zhou, Jihong Deng, Xiaojiang Zhan, Xiaoyang Wang, Yueqiang Wen, Ning Su, Xiaoran Feng, Qingdong Xu

**Affiliations:** 1 Department of Nephrology, Jiangmen Central Hospital, No. 23 Hai Bang Road, Jiangmen, 529000 China; 2 Department of Medical Statistics, Clinical Trials Unit, The First Affiliated Hospital, Sun Yat-Sen Univeristy, Guangzhou, China; 3 Department of Nephrology, The First Affiliated Hospital of Nanchang University, Nanchang, China; 4 Department of Nephrology, The First Affiliated Hospital of Zhengzhou University, Zhengzhou Univeristy, ZhengZhou, China; 5 Department of Nephrology, the Second Affiliated Hospital of Guangzhou Medical University, Guangzhou, China; 6 Department of Hematology, The Sixth Affiliated Hospital of Sun Yat-Sen University, Guangzhou, China; 7 Department of Nephrology, Jiujiang NO.1 People’s Hospital, Jiujiang, China

**Keywords:** Systemic immune inflammation index, Inflammation, All-cause mortality, Prognosis, Peritoneal dialysis

## Abstract

**Background:**

Chronic inflammatory disorders in peritoneal dialysis (PD) contribute to the adverse clinical outcome. Systemic immune inflammation index (SII) is the novel and convenient measurement that is positively associated with various diseases. However, scarce is known regarding the association between SII with all-cause mortality among PD patients.

**Methods:**

In this multi-center retrospective cohort study, 1,677 incident patients with PD were enrolled. Eligible patients were stratified into groups based on SII level: tertile 1(< 456.76), tertile 2(456.76 to 819.03), and tertile 3(> 819.03). The primary endpoint was the all-cause mortality. Both Cox regression analysis and competing risk models were used to examine the association between SII and all-cause mortality. Subgroup analysis was performed to assess the influence of the SII tertiles on all-cause mortality in different subgroups.

**Results:**

During the follow-up period of 30.5 ± 20.0 months, 26.0% (437/1,677) patients died, of whom the SII tertile 3 group accounted for 39.1% (171/437) of the deaths. Patients in the SII tertile 3 group had a higher all-cause mortality rate than patients in the SII tertile 1 and 2 groups (log-rank = 13.037, *P* < 0.001). The SII tertile 3 group was significantly associated with 80% greater risk (95% confidence interval:1.13 to 2.85; *P* = 0.013) compared with the SII tertile 1 group in multivariable Cox regression analysis. The competing risk model also indicated that the relationship between SII tertiles and all-cause mortality remains (subdistribution hazard ratio: 1.86; 95% confidence interval: 1.15 to 2.02, *P* = 0.011). Furthermore, the relationship between the log-transformed SII and all-cause mortality in patients with PD was nearly linear (*P* = 0.124).

**Conclusion:**

A close relationship was observed between the SII and all-cause mortality in patients undergoing PD, suggesting that more attention should be paid to the SII, which is a convenient and effective measurement in clinical practice.

**Supplementary Information:**

The online version contains supplementary material available at 10.1186/s12882-023-03451-4.

## Introduction

Currently, peritoneal dialysis (PD) accounts for approximately 11% of all dialysis patients worldwide [1]. The risk of mortality has increased in patients undergoing PD due to increasing risk for infections, malnutrition and cardiovascular diseases (CVD), which are to blame for approximately 40%–60% of all-cause mortality in the PD population [2]. Some traditional CVD risk factors [3] including smoking, physical activity, hypertension, diabetes, and hyperlipidemia, may contribute to poor survival outcomes among PD patients [4]. Other studies have shown that inflammatory status [5, 6], malnutrition [6–9], electrolyte disturbance [10–12], metabolic disorder [13–15], lower plant-based protein intake [16], alkaline phosphatases [17], and older age [18] were risk factors for all-cause mortality in patients undergoing PD. These factors above, particularly inflammation, are well-known nontraditional CVD risk factors [3] and may further increase the risk of death in such populations [19].

The pathogenic process of inflammation is typically complicated and multifactorial, involving both dialysis-related and dialysis-unrelated factors, which may lead to malnutrition, an adverse cardiovascular prognosis, and mortality in patients undergoing PD [20]. Certain inflammatory markers, such as C-reactive protein (CRP), neutrophil-to-lymphocyte ratio (NLR), and monocyte-to-lymphocyte ratio (MLR), which represent the systemic inflammatory response, are independent predictors of mortality in patients with PD [5, 18, 21]. Recently, a novel inflammatory marker, the systemic immune inflammation index (SII), which is inexpensive and easily accessible, has been a “hot issue”, and associated with cognitive decline [22], cerebrovascular diseases [23], atrial fibrillation [24], and poor prognosis of cancer [25]. Additionally, in the general population [23] of cardiovascular [26] or cerebrovascular disease patients [27], the correlation between SII and adverse overall survival has been found. A large, prospective, population-based study recently reported that compared with the lowest quantile of SII, the highest quantile of SII was associated with an approximate 24.6% greater risk of all-cause mortality. Thus, it may be as a useful marker of all-cause mortality in general population [23]. Moreover, there is a nearly linear relationship between log-transformed SII and all-cause mortality in patients with acute ischemic stroke [27], but a non-linear relationship between the log-transformed SII and all-cause death in patients with CVD [26, 28].

The SII may be better than other inflammation markers (such as CRP, NLR, and MLR) in clinical practice. First, as a new inflammatory biomarker, the SII can provide a more balanced and comprehensive assessment of immune and inflammatory responses [29]. As the SII level increases, inflammatory activity in various diseases may increase, leading to poor clinical outcomes [30]. Second, based on the counts of three types of circulating immune cells—neutrophils, platelets, and lymphocytes—the SII value reflects the inflammatory state and can present a better predictive value than the NLR and MLR [22, 31]. Third, compared with other inflammatory indicators such as CRP and interleukin-6 (IL-6), the SII can be easily obtained, routinely collected, and tested in hospitalized patients; thus, it might be useful in predicting poor prognosis.

Nevertheless, to the best of our knowledge, the association between the SII and all-cause mortality in patients undergoing PD has been elucidated only by a few papers. Therefore, we conducted this multi-center retrospective cohort study to test the hypothesis that the inflammatory status defined by the SII is associated with all-cause mortality in patients undergoing PD, ultimately laying the theoretical basis for its potential clinical assessment value.

## Material and methods

### Study population

**Fig. 1:**
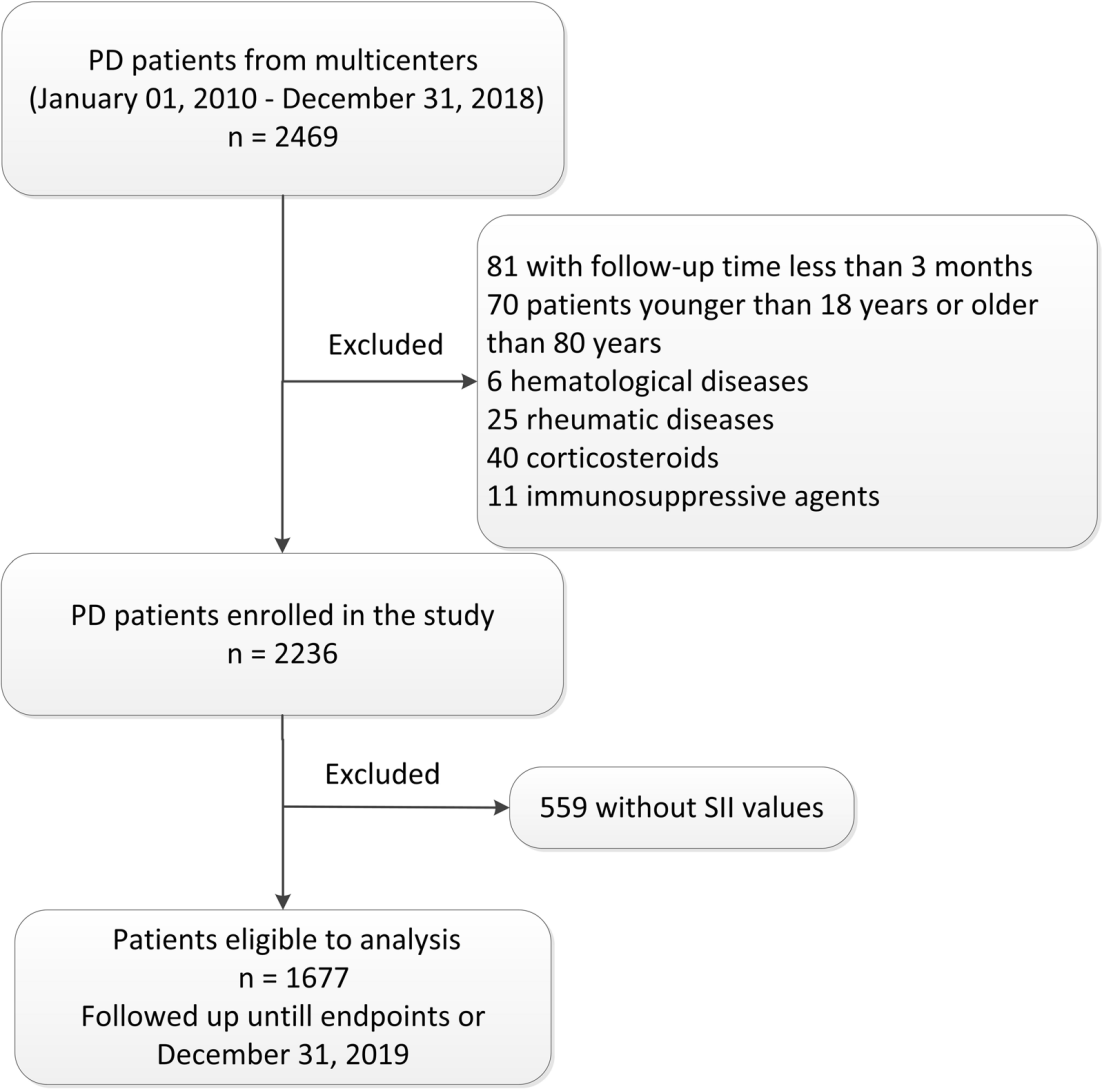
Flow chart of this study

A multi-center retrospective cohort study was conducted with 2,469 incident patients with PD between January 1, 2010, and December 31, 2018, who underwent PD treatment for at least 3 months at PD centers of six tertiary hospitals in China. Distributed in several provinces such as Guangdong, Shanghai, and Henan, each hospital has a specialized data registrar, follow-up management system, and electronic medical record system, and has over 400 patient undergoing PD. The main exclusion criteria were as follows: 1) patients with follow-up time less than 3 months (*n* = 81); 2) patients younger than 18 years or older than 80 years (*n* = 70); and 3) patients with a history of hematological diseases (*n* = 6), or rheumatic diseases (*n* = 25), with medication of corticosteroids (*n* = 40) or immunosuppressive agents (*n* = 11), as they could affect the lymphocyte and platelet counts. Patients without SII values were also excluded (*n* = 559). Finally, 1,677 patients were included in the study and followed up until the end of the study, or December 31, 2019. The flowchart is shown in Fig. 1. This study was approved by the investigational review board of Jiangmen Central Hospital (approval number 2022101) and was conducted in accordance with the Declaration of Helsinki in 2022. Written informed consent was not required because this retrospective study collected pre-existing hospital data.

### Data collection

All baseline data were collected 3 months after the initiation of PD therapy. Baseline demographic data included age; sex; current smoking and drinking habits; the presence of diabetes, hypertension, stroke, and cardiovascular diseases; and medication use, which was recorded based on the prescriptions. Clinical and biochemical data included blood pressure, white blood cells, hemoglobin, platelets, neutrophils, lymphocytes, serum creatinine, urea nitrogen, serum albumin, alkaline phosphatase, total cholesterol, triglycerides, sensitivity C-reactive protein (s-CRP), liver function, magnesium, calcium, phosphorus, and intact parathyroid hormone (iPTH). The SII was calculated as platelet count × neutrophil count/lymphocyte count. The baseline residual renal function was assessed using the Chronic Kidney Disease Epidemiology Collaboration creatinine equation. Total Kt/V was calculated using PD Adequest Software 2.0 (Baxter Healthcare Corp., Deerfield, Illinois, USA).

### Follow-up and study endpoint

The primary endpoint was the all-cause mortality rate. All patients were followed up until death, cessation of PD (switching to hemodialysis or renal transplantation), loss of follow-up, or the end of follow-up on December 31, 2019.

### Statistical analysis

The eligible patients were divided into three groups based on the tertile values of the SII. The lowest (Tertile 1), middle (Tertile 2), and highest (Tertile 3) SII tertile had an SII value of < 456.76, 456.76 to 819.03, and > 819.03, respectively. Continuous variables with a normal distribution were expressed as mean ± standard deviation, and those with a skewed distribution were described as median (25%–75% interquartile range [IQR]). Categorical data are expressed as frequencies or percentages (%). The SII was converted into logSII by the log transformation because of the non-normal distribution. Differences among the three groups were assessed using a one-way analysis of variance, the Kruskal–Wallis test, or χ^2^ test, as appropriate. Cumulative survival curves were generated using the Kaplan–Meier method and compared using the log-rank test. Cumulative incident curves were evaluated using the Fine and Gray method. The associations between the SII tertiles and all-cause mortality were evaluated using the Cox proportional hazards and subdistribution hazards models for competing risk analysis. To account for the non-linear correlation between the SII and all-cause mortality in patients with PD, we also employed a restricted cubic spline regression model to evaluate non-linearity.

We apply the method of “Computer Simulation Statistical Efficiency” to evaluate the sample size, which was performed using Empower(R) (www.empowerstats.com, X&Ysolutions, Inc., Boston, MA) [32]. The parameter settings are well documented according to the result of our study. The figure below shows the degree of research reliability corresponding to different sample size and odd ratio. The horizontal axis represents the sample size, the vertical axis represents the degree of reliability of the study. It can be seen that when the odd ratio value is 1.8 and the sample size is 1700, the test efficiency is over 0.95, which is already very reliable. Therefore, it can be considered that the sample size used in our article is appropriate.

### Subgroup analyses

To evaluate subgroup modification effects on the relationship between SII stratification and all-cause mortality, we conducted subgroup analyses of the association between SII stratification and all-cause mortality stratified by male and female, less than 65 years and older than or equal to 65 years, with or without diabetes, with or without hypertension, and those with or without a history of pre-existing CVD. The interaction between the SII tertiles and subgroups on all-cause mortality was examined using a formal interaction test.

SPSS statistical software version 22 (IBM Corp., Armonk, NY, USA) and R package 3.6.0 (https://www.r-project.org/) were used for the statistical analyses. Statistical significance was set at *P* < 0.05.

## Results

A total of 1,677 incident patients with PD were recruited and divided into three groups based on the tertiles of baseline SII value: the tertiles 1 (SII value < 456.79, *n* = 559), 2 (456.79 ≤ SII value ≤ 819.03, *n* = 559), and three groups (SII value > 819.03, *n* = 559). Among 1,677 patients, the mean age of the study population was 50.5 ± 14.0 years, 44.3% were female, and the percentages of hypertension, diabetes, and CVD history were 77.6%, 22.5%, and 23.4%, respectively. The mean follow-up period was 30.5 ± 20.0 months. At the end of this study, 26.01% (437/1,677) of patients had died, of whom the tertile 3 group accounted for 39.1% (171/437) of the deaths; 18.90% (317/1,677) of patients had undergone kidney transplantation and were transferred to hemodialysis; 3.64% (61/1,677) had been lost to follow-up; and the remaining 51.40% (862/1,677) of patients were still being followed up at these PD centers. Among the 437 deaths, 196 (44.8%) were attributed to cardiovascular mortality, 49 (11.2%) to cerebrovascular disease, 56 (12.8%) to infectious diseases, 9 (2.0%) to gastrointestinal bleeding, 11 (2.5%) to malignancy, 63 (14.4%) to other causes, and 53 (12.1%) to unknown causes.

### Comparisons of clinical parameters between groups

The baseline demographics and clinical and laboratory characteristics of the total study population after stratification by tertiles of SII values are summarized in Table 1. Patients in groups with a higher baseline SII level (tertile 3) compared with the reference group (tertile 1) tended to be male, older, and had higher levels of white blood cells, hemoglobin, platelets, neutrophils, monocytes, total cholesterol, triglycerides, calcium, and serum sCRP, but lower levels of lymphocytes, serum albumin, and iPTH (all *P* < 0.05). They also had a higher percentage of diabetes mellitus, angiotensin receptor blockers, statins, and aspirin use (all *P* < 0.05; Table 1).

**Table 1:** Baseline characteristics of individuals stratified by tertiles of SII

Variables	Total (*n* = 1677)	Tertiles 1 (*n* = 559)	Tertiles 2 (*n* = 559)	Tertiles 3 (*n* = 559)	*p* value
Sex, female(%)	743 (44.3)	276 (49.5)	237 (42.3)	230 (41.1)	0.010
Age(years)	50.5 ± 14.0	49.3 ± 14.4	50.3 ± 13.6	52.0 ± 14.0	0.005
Diabetes, n(%)	377 (22.5)	101 (18.1)	129 (23.1)	147 (26.3)	0.004
Hypertension, n(%)	1299 (77.6)	427 (76.7)	425 (76.2)	447 (80)	0.254
Stroke, n(%)	80 ( 9.4)	24 (10.2)	30 (10.4)	26 (7.9)	0.499
CVD, n(%)	199 (23.4)	52 (21.7)	59 (21)	88 (26.6)	0.203
Systolic pressure(mmhg)	144.0 ± 24.1	142.0 ± 22.5	145.3 ± 24.5	144.7 ± 25.2	0.081
Diastolic pressure(mmhg)	85.4 ± 15.1	85.5 ± 15.1	86.1 ± 14.6	84.6 ± 15.7	0.341
Medication					
CCB, n (%)	1221 (78.2)	395 (76.4)	410 (77.1)	416 (81.2)	0.124
ACEI, n (%)	81 (5.7)	28 (5.9)	21 (4.4)	32 (6.8)	0.265
ARB, n (%)	564 (38.2)	172 (35.4)	183 (36.6)	209 (42.7)	0.043
Statin, n (%)	219 (14.9)	53 (10.9)	76 (15.4)	90 (18.5)	0.004
Aspirin, n (%)	174 (12.0)	37 (7.7)	55 (11.3)	82 (17)	< 0.001
Laboratory variables					
White blood cell(10^9/L)	6.5 ± 2.3	5.1 ± 1.5	6.3 ± 1.7	8.1 ± 2.6	< 0.001
Platelet(10^9/L)	194.3 ± 80.5	142.8 ± 57.4	192.7 ± 59.7	247.3 ± 84.5	< 0.001
Hemoglobin(g/L)	89.4 ± 22.1	86.2 ± 22.5	90.2 ± 22.6	91.8 ± 20.8	< 0.001
Neutrophils(10^9/L)	4.5 ± 2.1	3.1 ± 1.0	4.3 ± 1.3	6.1 ± 2.3	< 0.001
Monocyte(10^9/L)	0.4 ± 0.2	0.4 ± 0.2	0.4 ± 0.2	0.5 ± 0.2	< 0.001
Lymphocyte(10^9/L)	1.3 ± 0.5	1.4 ± 0.6	1.3 ± 0.5	1.1 ± 0.4	< 0.001
Serum creatinine(umol/L)	788.4 ± 318.2	801.2 ± 323.8	797.6 ± 318.1	766.5 ± 312.0	0.133
Urea nitrogen(mmol/L)	22.4 ± 14.9	23.0 ± 9.6	22.1 ± 9.2	22.1 ± 22.2	0.526
Albumin(g/L)	36.0 ± 5.7	36.2 ± 5.5	36.3 ± 5.5	35.4 ± 6.1	0.016
Alkaline phosphatase(U/L)	84.6 ± 61.7	84.3 ± 73.3	82.3 ± 55.1	87.4 ± 55.0	0.374
Aspartate aminotransferase(U/L)	21.6 ± 15.1	22.1 ± 14.9	21.1 ± 16.4	21.7 ± 14.0	0.489
Alanine aminotransferase(U/L)	18.9 ± 24.5	20.3 ± 31.1	18.9 ± 23.7	17.5 ± 16.3	0.162
Total bilirubin(umol/L)	4.9 ± 3.1	5.0 ± 3.5	4.7 ± 2.6	5.0 ± 3.0	0.214
Cholesterol(mmol/L)	4.5 ± 1.3	4.3 ± 1.2	4.6 ± 1.4	4.7 ± 1.3	< 0.001
Triglyceride(mmol/L)	1.6 ± 1.2	1.4 ± 0.9	1.7 ± 1.6	1.6 ± 1.0	< 0.001
High density lipoprotein(mmol/L)	1.2 ± 0.4	1.2 ± 0.4	1.2 ± 0.4	1.2 ± 0.4	0.900
Low density lipoprotein(mmol/L)	2.8 ± 6.5	2.6 ± 1.0	3.1 ± 1.1	2.8 ± 1.0	0.380
iPTH(pg/ml)	198.0 (96.6, 351.4)	214.2 (114.3, 361.3)	199.4 (97.7, 352.5)	179.8 (68.1, 338.0)	0.017
Calcium(mmol/L)	2.1 ± 0.3	2.0 ± 0.3	2.1 ± 0.3	2.1 ± 0.3	< 0.001
Phosphorus(mmol/L)	1.8 ± 2.8	1.8 ± 0.5	2.0 ± 4.7	1.7 ± 0.6	0.364
Magnesium(mmol/L)	0.9 ± 0.4	0.9 ± 0.4	0.9 ± 0.3	0.9 ± 0.5	0.369
sCRP(mg/l)	3.2 (1.3, 8.5)	2.7 (1.2, 5.8)	3.0 (1.2, 6.5)	4.2 (1.6, 12.0)	< 0.001
Total Kt/V	2.3 ± 1.2	2.3 ± 1.0	2.4 ± 1.7	2.3 ± 0.9	0.420
RRF(mL/min/1.73m^2^)	4.1 ± 3.6	3.9 ± 3.2	4.2 ± 3.5	4.1 ± 3.9	0.416
SII (10^9/L)	611.1 (393.0, 963.0)	316.8 (240.3, 392.4)	610.9 (525.8, 710.4)	1214.0 (963.0, 1696.0)	< 0.001

*Abbreviations*: *CVD* cardiovascular diseases, *CCB* calcium channel blocker, *ACEI* angiotensin-converting enzyme inhibitor, *ARB* angiotensin receptor blocker, *iPTH* intact parathyroid hormone, *sCRP* sensitive C-reactive protein, *Total Kt/V* K, dialyzer clearance of urea; t dialysis time; V volume of distribution of urea, *RRF* renal residual function, *SII* systemic immune inflammation index

### Associations between SII and all-cause mortality

**Fig. 2:**
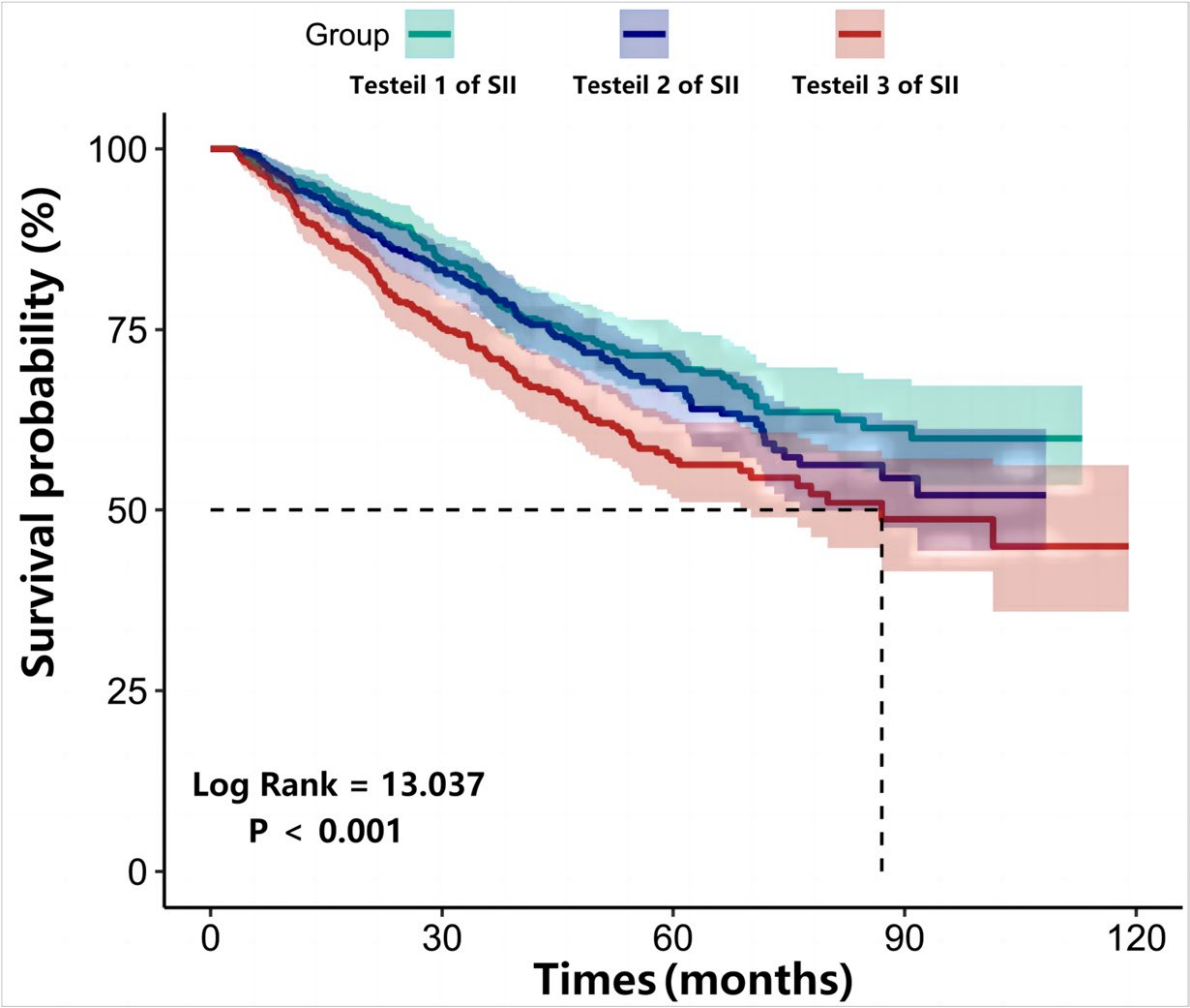
Kaplan–Meier curves for all-cause mortality stratified by SII

To confirm the association of the SII with all-cause mortality in the population, Cox regression analyses and a competitive risk model were utilized. Kaplan–Meier curves showed significant differences in all-cause mortality between the SII tertiles. Individuals in the SII tertile 3 group had a significantly higher death rate (SII tertile 3 group vs. SII tertile 2 group vs. SII tertile 1 group; 39.1% vs. 33.1% vs. 27.6%, respectively; *P* < 0.001, log-rank test; Fig. 2). In univariable Cox regression analyses, we found that compared with the lowest SII tertile group, the highest SII tertile group was associated with an approximate 54% greater risk of all-cause mortality (HR: 1.54, 95% CI: 1.21 to 1.95, *P* < 0.001). After a full adjustment for covariates, the association between SII tertiles and all-cause mortality in patients with PD remains (HR: 1.80, 95% CI: 1.13 to 2.85, *P* = 0.013). (Table 2).

**Table 2:** The associations of SII stratification with all-cause mortality in PD patients

Variables	Model 1(unadjusted)		Model 2(full adjusted)	
	HR or SHR(95%CI)	*p* value	HR or SHR(95%CI)	*p* value
All-cause mortality(Cox regression analysis)				
Tertles 1 of SII	Reference		Reference	
Tertles 2 of SII	1.16 (0.90, 1.48)	0.248	1.35 (0.82, 2.25)	0.241
Tertles 3 of SII	1.54 (1.21, 1.95)	< 0.001	1.80 (1.13, 2.85)	0.013
All-cause mortality(set switching to hemodialysis and kidney transplants as competitive risk)				
Tertles 1 of SII	Reference		Reference	
Tertles 2 of SII	1.16(0.90, 1.49)	0.242	1.48(0.88, 2.46)	0.136
Tertles 3 of SII	1.58(1.20, 1.94)	< 0.001	1.86(1.15, 2.02)	0.011

Model 1: unadjusted

Model 2: adjusted for age, sex, diabetes mellitus, hypertension, strokes, pre-existing cardiovascular disease, drug medication, total Kt/V, RRF, hemoglobin, urea nitrogen, Serum creatinine, albumin, cholesterol, triglyceride, High density lipoprotein, Low density lipoprotein, alkaline phosphatase, magnesium, calcium, phosphorus, iPTH, aspartate aminotransferase, alanine aminotransferase, total bilirubin

*Abbreviations*: *SII* systemic inflammatory index, *Total Kt/V* K, dialyzer clearance of urea; t dialysis time; V volume of distribution of urea, *RRF* renal residual function, *iPTH* intact parathyroid hormone, *HR* hazard ratio, *SHR* Subdistribution Hazard Ratio, *CI* confidence interval

**Fig. 3:**
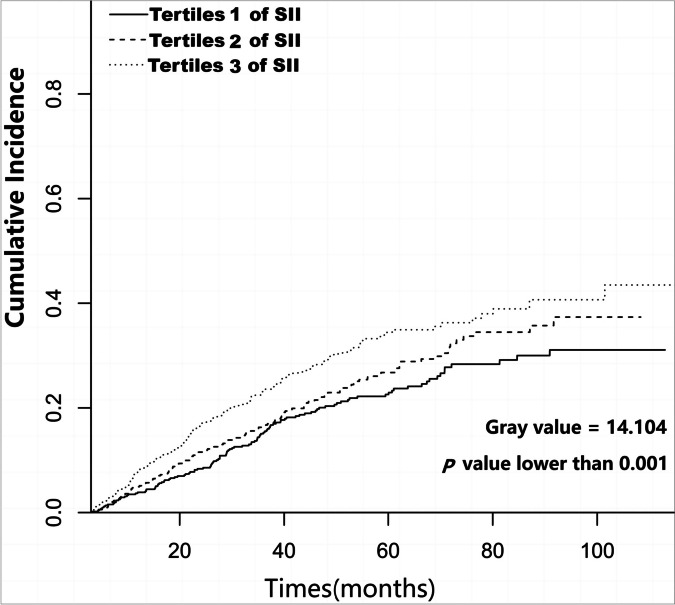
Cumulative incidence curves for all-cause mortality stratified by SII

In the Fine and Gray competing risk models, a higher SII was associated with a higher risk of all-cause mortality (gray value = 14.104, *P* < 0.001) (Fig. 3). After full adjustment, compared with the lowest SII tertile group, the highest SII tertile group was associated with an approximate 86% increased risk of all-cause mortality (SHR: 1.86, 95% CI: 1.15 to 2.02, *P* = 0.011). (Table 2).

Moreover, we used the cox regression model to analyze the relationship between CRP and mortality. The result showed that CRP > 3 mg /L was also correlated with mortality. However, when we adjusted for covariates, we found that CRP > 3 mg /L had nonsignificant effect on mortality, possibly due to the influence of CRP on mortality being influenced by other factors such as complications and nutritional status (Supplementary Table 1).

**Fig. 4:**
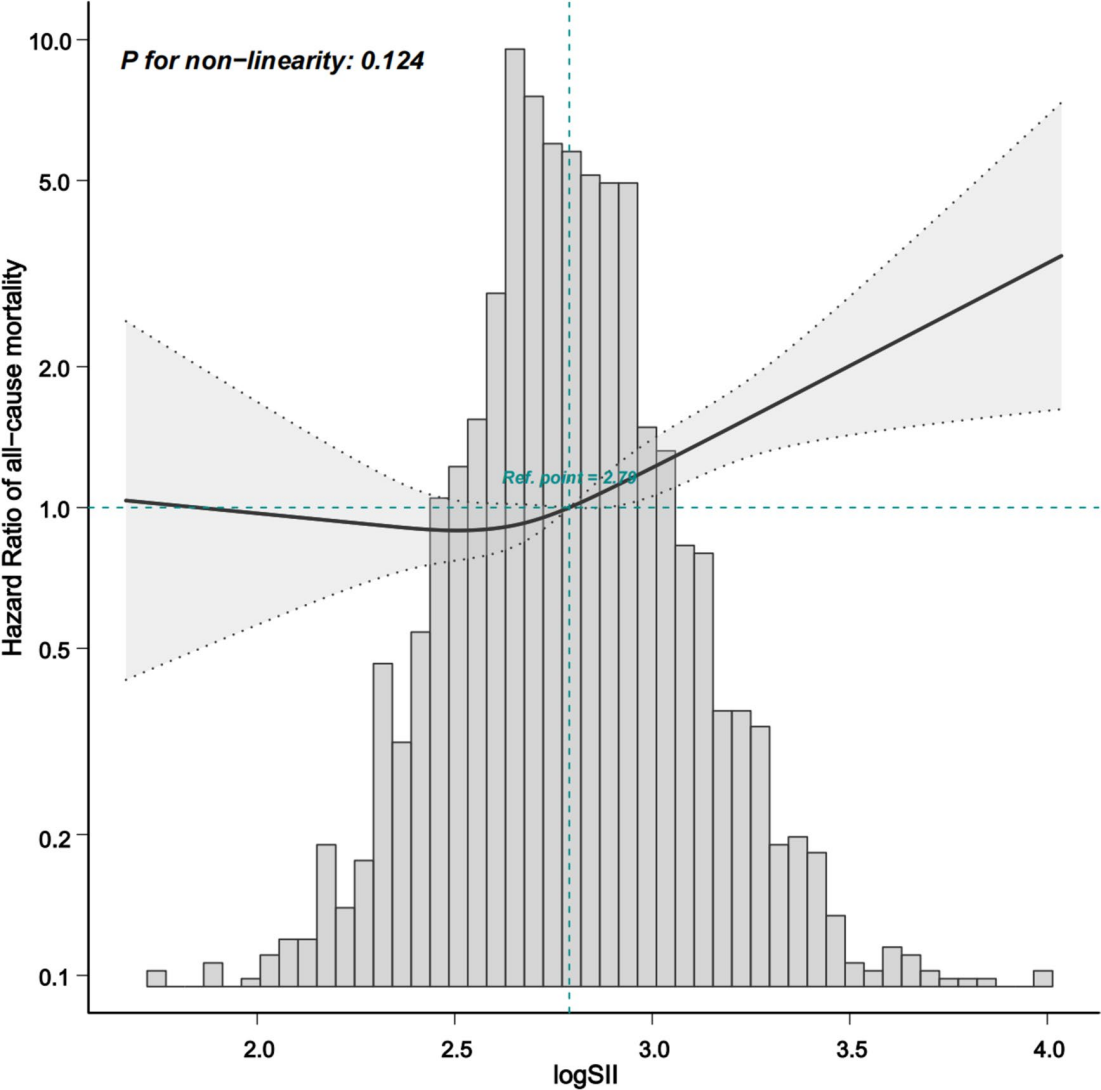
HR and 95% CI for the risk of all-cause mortality in PD patients along with the changes of logSII from the restricted cubic spline model

In addition, the restricted cubic splines showed that the risk of all-cause mortality increased gradually when log-transformed SII was > 2.78, while the risk of all-cause mortality remained unchanged when log-transformed SII was ≤ 2.78. The association between the log-transformed SII and all-cause mortality was nearly linear (*P* = 0.124, Fig. 4).

### Relationship between SII stratification and all-cause mortality in different subgroups

We investigated the association between SII tertiles and all-cause mortality in different subgroups, including male or female, less than 65 years and older than or equal to 65 years, with or without diabetes, with or without hypertension, and those with or without a history of pre-existing CVD. In the subgroup analysis, the results indicated that compared to the SII tertile 1 group, the SII tertile 3 group was associated with a 1.06 times greater risk of all-cause mortality for less than 65-year-old patients, 93% for those with hypertension, 4.62 times for those with diabetes, and 1.54 times for those without a history of pre-existing CVD (all *P* < 0.05, Table 3). However, these trends were not observed among patients aged 65 years or older, without hypertension, without diabetes, or with a history of pre-existing CVD. Moreover, no interaction was observed between sex, age, diabetes mellitus, hypertension, pre-existing CVD, or all-cause mortality.

**Table 3:** Subgroup analysis for the association of SII stratification and all-cause mortality in PD patients

Variables	Tertiles 1	Tertiles 2		Tertiles 3		*P* for interaction
		HR(95%CI)	*p* value	HR(95%CI)	*p* value	
**Age**						0.891
< 65 years	1.00	1.49 (0.79 ~ 2.82)	0.216	2.09 (1.16 ~ 3.76)	0.014	
≥ 65 years	1.00	1.07 (0.25 ~ 4.55)	0.929	0.92 (0.26 ~ 3.28)	0.894	
**Gender**						0.310
Male	1.00	0.84 (0.32 ~ 2.21)	0.723	1.49 (0.63 ~ 3.5)	0.362	
Female	1.00	1.7 (0.86 ~ 3.37)	0.129	1.88 (1 ~ 3.53)	0.052	
**Hypertension**						0.756
No	1.00	0.82 (0.44 ~ 1.51)	0.524	1.7 (0.97 ~ 2.99)	0.065	
Yes	1.00	1.67 (0.96 ~ 2.91)	0.071	1.93 (1.16 ~ 3.22)	0.011	
**Diabetes**						0.925
No	1.00	1.15 (0.63 ~ 2.07)	0.652	1.48 (0.85 ~ 2.59)	0.169	
Yes	1.00	1.14 (0.3 ~ 4.29)	0.846	5.62 (1.2 ~ 26.43)	0.029	
**Pre-existing cardiovascular disease**						0.251
No	1.00	1.53 (0.79 ~ 2.94)	0.207	2.54 (1.35 ~ 4.8)	0.004	
Yes	1.00	1.95 (0.66 ~ 5.75)	0.226	2.9 (0.88 ~ 9.56)	0.079	

*Abbreviations*: *SII* systemic inflammatory index, *Total Kt/V* K, dialyzer clearance of urea; t dialysis time, V volume of distribution of urea, *RRF* renal residual function, *iPTH* intact parathyroid hormone, *HR* hazard ratio, *CI* confidence interval; adjusted for age, sex, diabetes mellitus, hypertension, strokes, pre-existing cardiovascular disease, drug medication, t*otal Kt/V* RRF, hemoglobin, urea nitrogen, Serum creatinine, albumin, cholesterol, triglyceride, High density lipoprotein, Low density lipoprotein, alkaline phosphatase, magnesium, calcium, phosphorus, *iPTH* aspartate aminotransferase, alanine aminotransferase, total bilirubin

## Discussion

It is estimated that over 272,000 patients receive PD as a treatment for end-stage renal disease globally [1], and the number is increasing, with a considerable risk of adverse clinical prognosis. This study demonstrated that during the follow-up period of 30.5 ± 20.0 months, 437 (26.01%) patients died, which was consistent with other previous studies [33, 34], indicating that patients undergoing PD suffered an undesirable survival outcome. Thus, exploring the prognostic factors for survival in PD populations is crucial.

Calculated as (platelet count × neutrophil count/lymphocyte count), the SII is based on peripheral blood parameters that reflect the balance between inflammation and immunity [35, 36]. Growing evidence has been observed that the SII, as a novel inflammatory indicator, is correlated with a variety of adverse clinical outcomes [22–25, 31]. Notably, only a few studies focusing on the general population or patients with vascular disease have found that SII is independently correlated with all-cause mortality [23, 26–28]. Consistent with previous studies, our study showed an association between SII and all-cause mortality in patients undergoing PD and found that a higher SII corresponded to an increased risk of death in the PD population. Patients in the highest SII tertile had a significantly higher mortality rate, accounting for 39.1%. Compared with the lowest SII tertile group, the highest SII tertile group was associated with an approximately 80% greater risk of all-cause mortality, suggesting that the SII may act as a potential predictor of all-cause mortality in patients undergoing PD. Currently, this is a study applied by competitive risk model to focus on patients with PD to evaluate the value of SII in predicting all-cause mortality. Unlike other studies, our study applied competitive risk models and RCS curves to further demonstrate the relationship between SII and all-cause mortality in patients undergoing peritoneal dialysis. Althougt there exist several related studies, our manuscript is novel to a certain extent.

However, the underlying mechanisms by which the SII affects survival in these populations remain unknown and may be complicated. There are several possible explanations for these findings. One of the possible mechanisms may be the manifestation of systemic inflammation, whose prevalence ranges between 12 and 65% in PD populations [37], and which is a well-known risk factor for PD related mortality and plays an important role in poor prognosis [38]. SII is positively correlated with inflammatory indicators, including sCRP, NLR, and MLR, which have been regarded as prognostic biomarkers of poor survival in patients with PD [5, 6, 18–21]. Our study also found that patients in the groups with a higher baseline SII tended to have higher levels of serum CRP, which is a marker of inflammation and is significantly associated with a negative prognosis. Compared with composite indicators, such as NLR and MLR, a comprehensive indicator of neutrophils, platelets, and lymphocytes may be more stable, thereby increasing the application value of predicting the prognosis of patients with PD. A higher SII may represent a higher number of neutrophils and platelets, which stimulate the release of inflammatory mediators [39]. During inflammation, various pro-inflammatory cytokines and growth factors are released to stimulate the proliferation of macrophages, subsequently leading to the deterioration of body function [34]. Moreover, the higher the SII, the more severe the imbalance between inflammation and immunity and the more serious the inflammatory response [23].

Another potential mechanism might be the risk of malnutrition underlying the SII. Our study found that patients in groups with a higher baseline SII tended to have lower levels of serum albumin, which is a parameter representing nutritional status, indicating that SII may be related to nutritional status to a certain extent. Inflammation and malnutrition interact with and promote each other in dialysis patients [40], leading to poor overall survival.

In the subgroup analysis of patients over 65 years of age, the results showed no significance. We speculate that patients over 65 years of age are more susceptible to the majority of comorbidities and that the cause of death may not be exclusively inflammatory. Furthermore, our study indicated that, compared to the SII tertile 1 group, the SII tertile 3 group was associated with a 4.62 times greater risk of all-cause mortality in those with diabetes. Other study reported a relationship between SII and diabetes [41]. The inflammatory response is considered a potential mechanism involved in glucometabolic processes [41]. However, the underlying mechanism remains unclear.

The SII may be a prognostic marker of survival due to the following potential reasons. First, compared to neutrophil, platelet, or lymphocyte counts alone, the SII is relatively more stable and less likely to be changed by different pathological circumstances. Second, the SII integrates inflammatory response and immune regulation. Finally, the SII is a low-cost, easily obtainable and practical parameter; thus, it might be useful in predicting mortality risk among patients with PD.

It is noteworthy that several limitations of current research also exist. First, the present study is a retrospective study, thus, bias was inevitable. Second, we only collected baseline value of SII. Dynamically monitoring how SII changes is of highly necessity and worthy of further research. Third, we did not measure the markers of oxidative stress or inflammation, such as superoxide dismutase, malonaldehyde, IL-6, or tumor necrosis factor α, at baseline, which may also related with adverse prognosis in patients undergoing PD. Finally, due to the limitations of retrospective research, we did not collect sufficient clinical data on comorbidities scores. Also, we did not collect the characteristics of different dialysis centers.

## Conclusions

In summary, this study showed that systemic inflammation, defined by the SII, an inexpensive and easily accessible marker, may be a predictive factor for all-cause mortality in patients with PD. Improving the inflammatory status in patients with PD may provide clinical benefits for the long-term survival of the population; however, the mechanisms underlying SII are intricate and require further exploration.

### Supplementary Information

**Additional file 1:** **Supplementary Table 1.**

**Additional file 2: Supplementary Figure 1.** Sample size statistical efficiency. The figure shows the degree of research reliability corresponding to different sample size and odd ratio. The horizontal axis represents the sample size, the vertical axis represents the degree of reliability of the study. It can be seen that when the odd ratio value is 1.8 and the sample size is 1700, the test efficiency is over 0.95, which is already very reliable.

## Data Availability

The datasets used and/or analyzed during the current study are available from the corresponding author on reasonable request.
