# Supplementary material for: Association between systemic immune inflammation Index and all-cause mortality in incident peritoneal dialysis-treated CKD patients: a multi-center retrospective cohort study
Source: BMC Nephrol. 2024 Jan 3;25:8. doi: 10.1186/s12882-023-03451-4 (PMC10765751; doi:10.1186/s12882-023-03451-4)
Supplement: Supplementary file 1 — Additional file 1: Supplementary Table 1. [file 12882_2023_3451_MOESM1_ESM.docx]

**Supplementary table 1**

| Variable | **CRP(＞3mg/L)** | |
| --- | --- | --- |
|  | Model 1(unadjusted) | Model 2(full adjusted) |
| HR(95%CI) | 1.306(1.052,1.622) | 0.902 (0.611,1.333) |
| *P* value | 0.016 | 0.606 |

Adjusted for age, sex, diabetes mellitus, hypertension, strokes, pre-existing cardiovascular disease, drug medication, total Kt/V, RRF, hemoglobin, urea nitrogen, Serum creatinine, albumin, cholesterol, triglyceride, High density lipoprotein, Low density lipoprotein, alkaline phosphatase, magnesium, calcium, phosphorus, iPTH, aspartate aminotransferase, alanine aminotransferase, total bilirubin.

Abbreviations: *Total Kt/V*, K, dialyzer clearance of urea; t dialysis time; V volume of distribution of urea; *RRF* renal residual function; *iPTH*, intact parathyroid hormone.
