# Supplementary figures and images for: Association between systemic immune inflammation Index and all-cause mortality in incident peritoneal dialysis-treated CKD patients: a multi-center retrospective cohort study
Source: BMC Nephrol. 2024 Jan 3;25:8. doi: 10.1186/s12882-023-03451-4 (PMC10765751; doi:10.1186/s12882-023-03451-4)

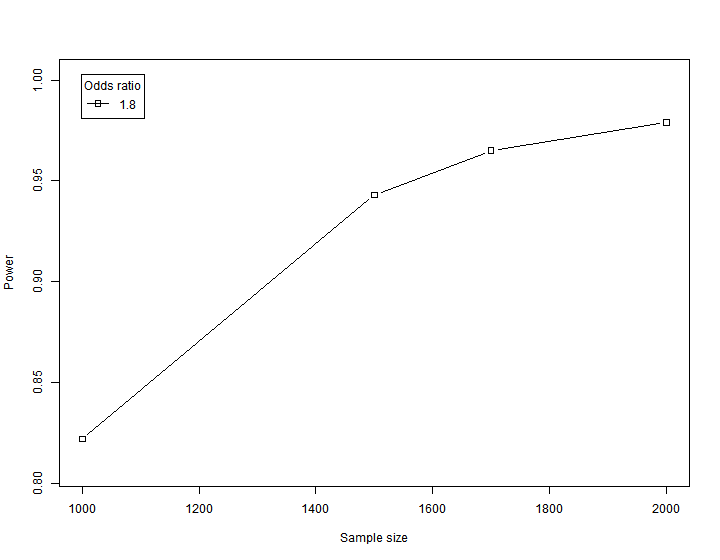

Supplement: Supplementary file 2 — Additional file 2: Supplementary Figure 1. Sample size statistical efficiency. The figure shows the degree of research reliability corresponding to different sample size and odd ratio. The horizontal axis represents the sample size, the vertical axis represents the degree of reliability of the study. It can be seen that when the odd ratio value is 1.8 and the sample size is 1700, the test efficiency is over 0.95, which is already very reliable. [file 12882_2023_3451_MOESM2_ESM.png]
